# Parthenogenesis in a captive Asian water dragon (*Physignathus cocincinus*) identified with novel microsatellites

**DOI:** 10.1371/journal.pone.0217489

**Published:** 2019-06-05

**Authors:** Kyle L. Miller, Susette Castañeda Rico, Carly R. Muletz-Wolz, Michael G. Campana, Nancy McInerney, Lauren Augustine, Celine Frere, Alan M. Peters, Robert C. Fleischer

**Affiliations:** 1 Department of Animal Care Sciences, Smithsonian’s National Zoological Park Washington, District of Columbia, United States of America; 2 Center for Conservation Genomics, Smithsonian Conservation Biology Institute, National Zoological Park, Washington, District of Columbia, United States of America; 3 George Mason University, Fairfax, Virginia, United States of America; 4 Saint Louis Zoo, One Government Drive, Saint Louis, Missouri, United States of America; 5 University of the Sunshine Coast, Sippy Downs, Queensland, Australia; Universidad de Cádiz, Facultad de Ciencias del Mar y Ambientales, SPAIN

## Abstract

Reptiles show varying degrees of facultative parthenogenesis. Here we use genetic methods to determine that an isolated, captive female Asian water dragon produced at least nine offspring via parthenogenesis. We identified microsatellites for the species from shotgun genomic sequences, selected and optimized primer sets, and tested all of the offspring for a set of seven microsatellites that were heterozygous in the mother. We verified that the seven loci showed high levels of polymorphism in four wild Asian water dragons from Vietnam. In all cases, the offspring (unhatched, but developed eggs, or hatched young) had only a single allele at each locus, and contained only alleles present in the mother’s genotype (i.e., were homozygous or hemizygous). The probability that our findings resulted from the female mating with one or more males is extremely small, indicating that the offspring were derived from a single female gamete (either alone or via duplication and/or fusion) and implicating parthenogenesis. This is the first documented case of parthenogenesis in the Squamate family Agamidae.

## Introduction

Parthenogenesis is a unique reproductive strategy in which females reproduce asexually in the absence of a male. Specifically, parthenogenesis occurs when an embryo develops from a female gamete without any contribution from a male gamete [1]. Vertebrates primarily reproduce after fusion of male and female gametes, and parthenogenesis, historically considered rare [2], is becoming a more commonly noted method of reproduction exhibited in an increasing number of species. In reptiles, the mode of reproduction is typically sexual [3]; however, obligatory parthenogenesis is known to occur in only one snake species, the Brahminy blindsnake (*Indotyphlops braminus*), and has been documented in over 20 species of lizards [3,4]. Reptilian facultative parthenogenesis has been most commonly recorded in isolated cases of captive specimens belonging to several Squamate families including Boidae, Viperidae, Elapidae, Acrochordidae, Colubridae, Pythonidae, and Varanidae [2–8]; however, Booth et al (2012) documented the first case of facultative parthenogenesis in wild populations of copperhead (*Agkistrodon contortrix*) and cottonmouth (*Agkistrodon piscivorus*) proving that it is not simply an unusual captive occurrence [9]. A 2018 publication by Allen et al. details molecular evidence for parthenogens produced from wild-collected elapid snakes [4]. This does not prove that these elapids were reproducing via parthenogenesis in the wild; however, it does confirm that wild-collected reptiles can be capable of parthenogenesis and further illustrates that it’s not just an anomaly exhibited in captive-born/raised individuals.

Unisexual reproduction is found in less than 0.1% of all vertebrate species [10]. Unisexual reproduction falls into three categories: hybridogenesis (hemiclonal reproduction where only the female half of the genome is passed to the offspring following fertilization), gynogenesis (reproduction in the presence of sperm with no fertilization of the egg), and true parthenogenesis (no requirement from male) [11]. In cases of vertebrate facultative parthenogenesis multiple mechanisms are proposed. Terminal fusion automixis is the dominant mechanism proposed for facultative parthenogenesis in vertebrates [1,9,12,13]. In terminal fusion automixis, heterozygosity is restricted to the tips of the chromosomes and genetic signatures of randomly screened microsatellite loci tend to demonstrate elevated homozygosity. Nonetheless, gamete duplication and spontaneous development of a haploid individual from an unfertilized egg also result in complete homozygosity [14–16].

The Smithsonian’s National Zoological Park (NZP) houses a lone female Asian Water Dragon (*Physignathus cocincinus* NZP Accession Number 307165 [code WD-10]) that was captive hatched July 5, 2006 at the St. Louis Zoo. This animal was acquired by NZP on November 8, 2006, and housed either individually or with other females prior to reaching sexual maturity. Records of this lizard producing clutches of eggs date back to March 2009; however, zoo staff assumed clutches were infertile and did not artificially incubate. After several reports of parthenogenesis and sperm storage in other reptile species came to the attention of NZP staff, a clutch of seven eggs laid on 14 November 2015 were artificially incubated. Eggs were fertile, noted by embryonic development and veining that was evident when candling the eggs. None of the eggs from this clutch produced living offspring, but after 70 days of incubation fully developed hatchlings were found inside two of the eggs. The following clutch, laid 10 April 2016 was also artificially incubated, found to be fertile, and although most eggs died during various times during development, one fully developed offspring (WD-11) hatched with assistance from zoo staff on 16 June 2016. A clutch laid on 14 September 2018 also produced one viable offspring that hatched on 21 November 2018 (NZP accession number 307726). WD-11 and 307726 are the only living offspring from WD-10. All artificially incubated clutches collected since November 2015 have contained fertile eggs, but embryonic mortality typically occurs in the early stages of development or fully formed offspring are found dead inside the egg after the suggested incubation period of 60–75 days. WD-11 was confirmed to be a female upon development of secondary sex characteristics at 18 months of age. 307726 is still too immature to identify sex.

Here we report on genetic analysis designed to determine whether this female water dragon (WD-10) produced offspring asexually and what mechanism was responsible. An alternate hypothesis is that this animal stored sperm from a prior mating, which is highly unlikely given the isolation from males. To the authors’ knowledge, this is the first documented case of parthenogenesis in the family Agamidae, which brings insight into the evolutionary history of parthenogenesis in reptiles and more specifically Squamate lineages.

## Methods

### Sample collection

We collected one blood sample from the mother (WD-10) during a routine veterinary visit. For offspring (WD-1-8), we used skin tissue from embryos within eggs laid on 10 April 2016 containing offspring that died at varying time points in development prior to viability. A buccal swab was collected from the living offspring WD-11. NZP herpetology staff artificially incubated eggs for a minimum of 60 days before opening them to assess status of the embryos. All samples from live animals were collected opportunistically in coordination with routine exams; thus NZP IACUC approval was not required.

We requested and received tissues from the Ambrose Monell Cryo Collection (AMCC) at the American Museum of Natural History (AMNH) for four wild Asian water dragons (Table 1) to validate that microsatellites were polymorphic and heterozygous in wild individuals of the species.

**Table 1:** Metadata on four wild Asian water dragon samples obtained from collections at the American Museum of Natural History.

AMCC ID#	Dept/Partner ID	Collection Date	Prep Type	Country	State	County
106643	R-148566	05/22/2000	Fluid	Vietnam	Ha Giang	Vi Xuyen
141239	R-503347	0/18/2004	Tissue	Vietnam	Lao Cai	Van Ban
192724	R-500135	05/26/2010	Tissue	Vietnam	Quang Nam	Nam Giang District
192902	R-500155	06/01/2010	Tissue	Vietnam	Quang Nam	Phuoc Son District

### Molecular procedures

We extracted DNA from Asian Water Dragon samples using the Qiagen Blood & Tissue Kit (Germantown, MD) following the manufacturer’s instructions. DNA concentrations were determined using a Qubit 2.0 fluorometer (ThermoFisher Scientific) with a dsDNA high sensitivity kit.

We tested 17 microsatellite primer sets developed for the Eastern Water Dragon, *Intellagama lesueurii* [17]. We were able to successfully amplify Asian Water Dragon DNA with 7 primer sets, but only one (EWD69) showed polymorphism. We used Qiagen’s Multiplex PCR kit in the following recipe: DNA template (10–25 ng/ul), 5 μl of Qiagen Multiplex PCR mix, 1 μl of primer mix (2 μM each primer). Cycling conditions consisted of denaturing at 94 °C for 15 min; 30 cycles at 95 °C for 30 s, 58° C for 1.5 min, and 72 °C for 1.5 min; with a final extension of 72 °C for 10 min.

To identify novel microsatellites, we sequenced genomic DNA using an Illumina MiSeq with a 600-cycle Reagent Kit v3 (2 × 300 bp). We generated 300 bp paired-end reads for three water dragons (the mother WD-10, and two offspring: WD-4 and WD-5). Each individual was tagged with two unique barcodes for demultiplexing using the BaseSpace pipeline (Illumina, Inc.). Library read qualities were checked using FastQC 0.11.4 [18]. Reads were trimmed using Trimmomatic 0.36 (options: ILLUMINACLIP:NexteraPE-PE.fa:2:30:10, LEADING:3, TRAILING:3, SLIDINGWINDOW:4:20, MINLEN:36) [19]. We merged trimmed reads with FLASH 1.2.11 (option: -M 301) [20] and removed PCR duplicates using CD-HIT-DUP 0.5 [21]. Tri- and tetra-nucleotide microsatellites were mined from the deduplicated sequences and corresponding PCR primers for these loci were designed using MSATCOMMANDER 1.0.8-beta under default settings [22]. In total, we identified 52,864 microsatellites from 2,785,132 merged sequences (1.9%). Read statistics are summarized in Table 2.

**Table 2:** Total number of raw and merged reads per individual. Merged reads were used in the analyses to identify microsatellites. Raw reads were accessioned in the NCBI Sequence Read Archive (SRA).

Individual ID	Status	Raw Read Pairs	Merged Sequences	SRA Accession #
WD-10	Mother	3303890	1618164	SRR6889000
WD-4	Offspring	1386919	577283	SRR6888999
WD-5	Offspring	1288234	589685	SRR6889001

We also attempted to assess heterozygosity of these three individuals using the shotgun sequencing data. The merged reads were aligned against the Central bearded dragon (*Pogona vitticeps*) genome (assembly pvi1.1) [23] using BWA-MEM 0.7.15-r1140 [24]. PCR duplicates were removed using SAMtools 1.3.1 rmdup [25]. Variants were called using SAMtools mpileup (options -C50 -t DP,AD) and BCFtools 1.3.1 call (options -m -v). Sequencing depth was too low to permit robust genotyping. Of the 239,922 variants (210339 single nucleotide variants, 29583 indels) identified against the *Pogona* genome, 219,843 had a total depth (across all three individuals) of 1 and 18,693 had a total depth of 2. No informative sites remained after filtering for a minimum sequencing depth of 10 and a minimum quality of 30 using VCFtools 0.1.15 [26]. Thus the shotgun sequence data were uninformative for assessing parthenogenesis.

We selected 14 microsatellite primer pairs for further development based on their completeness, array length (>20 repeats) and product size. We tested for PCR amplification success and polymorphism using DNA from the mother and four offspring. PCR conditions were carried out in a 25 μl reaction containing 3 μl of DNA, 2.5 μl of 10× Amplitaq Gold PCR buffer, 0.4 μM of each primer, 0.2 mM of DNTPs, 1.5 mM of MgCl_2_, and 0.15 U of AmpliTaq Gold DNA polymerase (Applied Biosystems). PCR conditions consisted of denaturing at 95 °C for 10 min; 30 cycles at 95 °C for 15 s, 56° C for 30 s, and 72 °C for 30 s; with a final extension of 72 °C for 5 min. PCR products and Generuler ultralow range ladder (Thermo Scientific) were separated on an 3% agarose gel for 2 h at 70 V and stained using GelRed. Six of these primer pairs were heterozygous in the mother.

All samples from the National Zoo and AMNH collection were amplified with the six new primer sets (Table 3) as well as the primer set (EWD69) from Frere et al. (2012) [17]. Forward primers were labeled with FAM or HEX fluorescent labels. We followed the PCR protocol as outlined above for testing the 7 microsatellites developed for *Intellagama lesueurii*, and processed them on an Applied Biosystems 3130xl DNA Analyzer with GeneScan ROX 500 ladder, and analyzed with GeneMapper v5.0 (ThermoFisher). Allele calls from GeneMapper were standardized by hand when necessary.

**Table 3:** Details of the six microsatellites developed from Illumina shotgun sequence data that were heterozygous in female WD-10 Asian Water Dragon (*Physignathus cocincinus*). We used the shotgun library sequence to estimate the size of the amplicon product.

Locus name	Forward primer (5'-3')	Reverse primer (5'-3')	Estimated size	Repeat motif	SRA Accession #
Pcoc2	GCTTTGTTGCCGTAGAGTGG	AAGGTCATCTGGTCTCAAGAAC	211	(AGAT)_25_	SRR6889000.76842
Pcoc6	GAGCCATACACAGGACCTTG	CTCACGAATGCTGAGAAGGC	198	(ACAT)_22_	SRR6889000.137347
Pcoc7	GGCATTCCATTAGAGAGGCAG	ACAGTCCATGGGCAATTCTG	162	(AGAT)_26_	SRR6889000.176808
Pcoc9	TGTTCAGACTATGGATGCAGC	TGGCACATTATGTCCCAACC	281	(AGAT)_22_	SRR6889000.488305
Pcoc10	TCCCATTCATACTCCGGTCC	AGATGCCCAGAGACTCTTGG	230	(AATC)_20_	SRR6889000.579750
Pcoc13	TGGGCACAGTGATGTCTAGG	TGCCCAGAGTAGTGCTTCAG	218	(AGAT)_27_	SRR6889000.941431

## Results and discussion

In total seven microsatellite loci (Table 3) were scored for each individual (Table 4). As noted above, we used the seven loci because the mother Asian Water Dragon (WD-10) was heterozygous for all seven (but not for others we tested). The seven loci showed high levels of polymorphism in four wild Asian Water Dragons from Vietnam (mean H_obs_ of 0.786). These four animals were collected in separate localities within Vietnam (Table 1). Thus our genotype calls were “homozygous”, but the offspring could also have been “hemizyogous”, depending on whether there was fusion to form diploid complements, or direct development of single haploid gametes (less likely, but possible). Either way, these results unambiguously support the hypothesis that the embryos produced by WD-10 were of parthenogenetic origin and not due to sperm storage and latent fertilization (or they would have been heterozygous at most or all loci because of differing paternally derived alleles).

**Table 4:** Genotype data at seven microsatellite loci for 14 Asian Water Dragons. WD-1-11 are a mother Asian water dragon housed at the National Zoo, eight of her unhatched offspring, and a single hatched and living offspring. Asian water dragons from AMNH AMCC were also screened to confirm polymorphism of the microsatellite loci in four animals collected in Asia.

ID	Sample type	EWD69		Pcoc2		Pcoc6		Pcoc7		Pcoc9		Pcoc10		Pcoc13	
WD-10	Mother	304	316	212	224	200	204	162	170	312	324	212	236	222	234
WD-1	Offspring	316		212		204		170		312		212		222	
WD-2	Offspring	316		224		200		162		312		236		222	
WD-3	Offspring	316		212		200		162		324		236		234	
WD-4	Offspring	316		224		200		170		324		212		234	
WD-5	Offspring	304		224		200		missing		324		212		234	
WD-6	Offspring	304		212		200		170		312		212		222	
WD-7	Offspring	316		224		200		170		324		212		234	
WD-8	Offspring	316		212		200		162		324		212		234	
WD-11	Living offspring	316		212		200		170		324		212		222	
106643	Wild	338		187		204		162	170	300	308	226		174	178
141239	Wild	328		200	208	196	208	154	178	320	332	222	230	178	182
192724	Wild	312	320	216	220	174	182	170	174	288	296	212	232	178	206
192902	Wild	304	308	200	210	200	202	158	170	292	312	222		216	220

The hypothesis of parthenogenetic origin for the embryos is also supported probabilistically. Since we observed only homo- or hemizygous genotypes for both alleles for all markers in offspring, the maximum probability of this observation by chance arises in a source population of males in which all loci are biallelic with equal frequencies. In this case, each random mating produces half homozygotes and half heterozygotes since the female is heterozygous for all alleles. Therefore, the maximum probability of 62 unlinked homozygous observations (63–1 missing genotype) is 0.50^62^ = 2.17 × 10^−19^. Even if we assume that all alleles are perfectly linked (an overly conservative assumption since recombination is observed in our data), the maximum probability is still significant at an alpha of 0.01 (p = 1.95 × 10^−3^ for 9 independent observations).

The female was most likely facultatively parthenogenetic, although we do not have evidence that she can mate and breed sexually. Two types of facultative parthenogenesis have been proposed: systematic and accidental [3]. Accidental parthenogenesis is associated with a low hatching rate that has several plausible explanations described by Shibata et al, one of which is that it occurs as a form of emergency reproduction in response to isolation from mates [3]. Van der Kooi & Schwander propose that facultative parthenogenesis in vertebrate species that primarily reproduce sexually are reproductive errors; this supports the idea of accidental parthenogenesis and may also account for the low hatchling viability rate [27]. Alternatively, it may be adaptive to have this capability under certain environmental conditions (perhaps because of high dispersal leading to situations where males may not occur). Out of 64 eggs recovered from WD-10, only two hatched resulting in viable offspring WD-11 and 307726. This is a hatch rate of 3.125%. NZP herpetology keeper records indicate that at least 30 of the eggs recovered were fertile; thus the fertility rate is approximately 46.875%. With so few offspring resulting from an almost 50% fertility rate, accidental parthenogenesis is a reasonable explanation; however, this could also indicate that other factors may be at play preventing the offspring from developing properly. There is no evidence to suggest this occurred with any of WD-10’s eggs, but imperfect incubation parameters such as temperature and humidity inconsistencies can impact development. Future study may give us more insight into what specifically is causing this result.

Mechanisms by which the mother could produce complete homozygosity through parthenogenesis in offspring include terminal fusion automixis [1,9,12], gamete duplication [15], and spontaneous development of a haploid individual from an unfertilized egg [16]—as discussed in Dudgeon et al. [13]. Terminal fusion automixis is the dominant mechanism proposed for facultative parthenogenesis in vertebrates [1,9,12,13]. We have no evidence for any particular mechanism from our analyses, but it is worth noting that no viable haploid offspring were reported in Portnoy’s 2014 study, only stillborns; however, parthenogenic offspring WD-11 and 307726 are still thriving and in perfect health. More analysis of asexual reproduction in Squamate lineages is needed to fully understand the mechanisms responsible, the environmental factors that trigger it, and its potential evolutionary advantage.

Given all documented reports to date, it is safe to suggest that facultative parthenogenesis in reptiles is not as rare as was once thought with more cases proven every year, and it is unlikely that the successive clutches of parthenogenic clutches produced by WD-10 were the result of reproductive error. It is interesting to note that it may not have been unusual for WD-10 to facultatively produce clutches across seasons either, as successive virgin births with low levels of viability were also described in checkered garter snakes (*Thamnophis marcianus*), Colombian rainbow boas (*Epicrates maurus*), and common boas (*Boa constrictor imperator*) (although these species are ovoviviparous) [7,28,29]. The low viability of facultatively produced parthenogens may support that this method is a reproductive error [27]; however, facultatively produced parthenogenic clutches of several species of boids and pythonids in captivity have proven to produce large numbers of highly viable offspring [8]. Further study may give us the opportunity to explore if WD-10 can produce sexually and the reproductive viability of her offspring WD-11 and 307726. It will be especially interesting if WD-11 and/or 307726 facultatively reproduce viable eggs.

## Conclusion

The study of the origin and evolution of parthenogenesis has greatly benefited from advancements in genetic methods and the utility of captive collections to provide opportunities to test these hypotheses. The Asian water dragon reported here demonstrates a fairly simple beginning to contributing to a more complex question and we hope this article will encourage other zoological institutions to incubate eggs laid by female reptiles in the absence of mates to assess their parthenogenic capabilities; thus adding to the available knowledge of what was once thought to be a rare reproductive event only noted in random captive accounts.
